# In vitro propagation and cytological analysis of *Sophora mollis* Royle: an endangered medicinal shrub

**DOI:** 10.1186/s43141-021-00140-3

**Published:** 2021-03-15

**Authors:** Aakriti Bhandari, Harminder Singh, Amber Srivastava, Puneet Kumar, G. S. Panwar, A. A. Mao

**Affiliations:** 1 Botanical Survey of India, Northern Regional Centre, Dehradun, Uttarakhand 248195 India; 2 Botanical Survey of India, Eastern Regional Centre, Shillong, Meghalaya 793003 India; 3 Botanical Survey of India, Kolkata, West Bengal India

**Keywords:** Micropropagation, Shoot tip culture, Plant growth regulators, *Sophora mollis*, Chromosome number

## Abstract

**Background:**

*Sophora mollis* Royle (family Fabaceae, subfamily-Papilionaceae) is a multipurpose legume distributed in plains and foothills of the North-West Himalaya to Nepal and is facing high risk of extinction due to habitat loss and exploitation by the local people for its fuel and fodder values. Therefore, the present study was conducted to standardize a micropropagation protocol for *Sophora mollis* by using shoot tip explants and to study the meiotic chromosome count in the species.

**Results:**

Multiple shoots were induced in shoot tip explants of *Sophora mollis* in Murashige and Skoog medium supplemented with different concentrations of cytokinins alone (BAP, TDZ, and Kinetin) and in combination with varying concentrations of NAA. MS medium supplemented with BAP (8.9 μM) was observed to be the optimal medium for multiple shoot induction and maximum 25.32 shoots per explant was obtained with average length of 4.5 ± 0.8 cm. In vitro developed shoots were transferred onto rooting media supplemented with different concentrations of auxin (IAA, IBA, and NAA). Maximum 86% rooting was observed in half-strength MS medium supplemented with 21.20 μM NAA with an average of 21.26 roots per culture. In vitro raised plantlets were adapted to greenhouse for better acclimatization and 60% plants were successfully transferred to the open environment. Based on the chromosome counts available from the literature and the current study, the species tend to show a basic chromosome number of *x* = 9.

**Conclusion:**

The micropropagation protocol standardized can be helpful for the ex situ mass multiplication and germplasm conservation of the endangered species. Moreover, the ex situ conservation approach will be helpful in actively bridging the gap between ex situ and in situ approaches through the reintroduction of species in the wild. The cytological studies revealed the basic chromosome number *x* = 9 of the species.

## Background

*Sophora mollis* (Royle) Baker belongs to family Fabaceae (subfamily-Papilionaceae) is a small deciduous perennial shrub with dense hairy twigs and yellow flowers generally blooms in the month of March to May [1]. It is commonly known as peeli sakina, and distributed to semi-exposed to shaded moist slopes of forest edges in the Western Himalaya at an altitude range of 700-1500 m in India (Jammu and Kashmir, Himachal Pradesh, and Uttarakhand), Pakistan, Afghanistan, and China [2]. Due to its continuous exploitation from wild habitats by the local people to fulfill their needs, embark it into endangered category as per its conservation status [3].

Previous studies revealed various pharmacological and therapeutic properties of this genera and extensively being used in traditional Chinese drugs since time immemorial. Genus *Sophora* is a source of more than 300 compounds such as quinolizidine alkaloids (matrine and oxymatrine) and flavonoids (prenylated and isoprenylated flavonoids). Phytochemical constituent of genus *Sophora* is medicinally used as anti-cancerous, anti-asthmatic, anti-neoplastic, anti-microbial, anti-pyretic, cardiotonic, anti-inflammatory, diuretic, skin diseases like eczema, colitis, and psoriasis. Besides their curative properties they also have antioxidant properties [4]. *Sophora mollis* is also a rich source of phytochemical constituents and eight chemical compounds, viz., (*E*)-phytyl epoxide, 7,11,15-trimethyl-3-methylenehexadecane-1,2-diol, loliolide, scopoletin, hexacosanol, octacosanol, β-sitosterol, and daucosterol have been isolated from the aerial part of it [5].

*S. mollis* is mainly propagated through seeds and root suckers in the wild but regular cutting of the species for fodder purpose has resulted into poor seed setting and has badly affected the regeneration potential of the species in wild. This might be one of the probable reasons of its population shrinkage from the wild and it is further compounded by various other factors such as habitat degradation, forest fire, and climate change. To overcome the challenges of conventional propagation methods, new advance method of micropropagation is considered as an effective tool for ex situ conservation and its perpetuation in the wild. In vitro morphogenesis seems to be difficult to achieve in the Papilionaceae but with few exceptions; micropropagation protocol were successfully standardized for the *Robinia pseudoacacia* [6], *Pterocarpus indicus* [7], and *Cassia senna* [8], species phylogenetically close to *Sophora*. In vitro studies were also conducted in some species of *Sophora*, *viz.*, *S. toromiro* [9], *S. tonkinensis* [10], and *S. flavescens* [11] and in vitro propagation protocol were successfully standardized for them. But best of our knowledge, there is not even a single study on in vitro propagation of *S. mollis*.

*S. mollis* has a scattered distribution over a wide range from Central Asia to foothills of Western Himalayas and beyond. However, chromosome counts are available from very few regions. Previously meiotic chromosome number (*n* = 9) has been reported in *S. mollis* from India [12, 13], Iran [14–16], and Russia [17]. Bir and Kumari [12] reported 2*n* = 18 in the species from Sangam, Panchmari, Madhya Pradesh, India, with normal meiotic behavior. Noori et al. [15] worked out mitotic chromosome count in the species (2*n* = 18) with mean chromosome size range between 1.40-2.40 μm. Advance genomic studies in the genus showed that the species existed at diploid (2*x*) level with 2.04 pg genomic size 2C DNA amount [16]. The basic chromosome number varies in the genus *Sophora* which is a paraphyletic group of species having basic chromosome numbers, i.e., *x* = 7, 8, 9, 11, 14 [15].

Therefore, this study was undertaken to develop an efficient micro-propagation protocol for the mass multiplication of *S. mollis*, cytological analysis of the species and to rehabilitate the species in the wild.

## Methods

### Initiation of aseptic culture and shoot induction

The young terminal shoot tip explants (2.0-3.5 cm) of *S. mollis* were collected from the plants conserved in the Experimental Botanical Garden and identified with the help of herbarium specimen (BSD 123495) for the further in vitro and cytological studies. Explants were initially washed under running tap water for 30 min followed by Tween-20 (Himedia Laboratories, Mumbai, India) to remove dirt particles, traces of soil, and followed by fungicide treatment (1% bavistin) for disinfection of the explants for 30 min. Thereafter, explants were disinfected with different surface sterilizing agents, i.e., ethanol (70%), sodium hypochlorite (6%) (Merck & Co., USA) and mercuric chloride (0.1%) (Himedia Laboratories, Mumbai, India) with different time duration of 2, 5, and 8 min, respectively. After each treatment, explants were washed thrice with sterilized double distilled water. Properly disinfected shoot tip explants were further used for the organogenesis experiments. The pH of the medium was adjusted to 5.7 before autoclaving at 121 °C for 15 min and then sterilized shoot tip explants were inoculated onto basal MS medium [18] supplemented with agar (6%), sucrose (30 g l^−1^), and various PGRs alone or in combinations. Cultures were maintained in the culture room at 24±2 °C, under a 16/8 h light and dark cycle with a light intensity of 47.29 μmol m^−2^ s^−1^ provided by white fluorescent PAR lights (40 W; Wipro, India). All the plant growth regulators (PGRs) applied was procured from Himedia Laboratories, Mumbai, India, and glass wares used (conical flask: Borosil 4980, 250 ml, and 85×140 mm; culture tubes: 38×200 ml) were from Borosil, India.

Explants (size, 1.5-2.0 cm) were inoculated onto basal MS medium (control) and MS medium augmented with different concentrations of cytokinins, viz., 6-benzylaminopurine (BAP) (2.2 to 11.1 μM), N-phenyl-N′-1,2,3-thiadiazol-5-yl urea (thidiazuron/TDZ) (2.27 to 6.8 μM), and kinetin (2.32 to 9.3 μM). Subsequently, the optimal concentration of BAP (8.9 μM), TDZ (4.54 μM), and kinetin (6.9 μM) were further tested in combination with different concentrations of naphthalene acetic acid (NAA) (0.53-2.65 μM) to observe the synergistic effect of both the PGRs on shoot induction and proliferation.

After shoot initiation, shoot proliferation was performed in MS medium supplemented with BAP (8.9 μM) having 0.5% agar (Himedia Laboratories, Mumbai, India). Shoot proliferation cultures were sub-cultured at regular interval of 3 weeks. Shoot multiplication rate was calculated on the basis of percentage of explants with positive response, number of total shoots per explant and shoot height after 8 weeks of incubation.

### Root induction

In vitro developed single shoots/shoot cluster of 2-3 cm length were inoculated onto MS and modified MS medium (half strength and quarter-strength). Further, half-strength MS medium supplemented with different concentrations of auxins, viz., indole-3-acetic acid (IAA) (5.71, 11.42, 17.13, and 22.84 μM), indole-3-butyric acid (IBA) (4.9, 7.36, 9.8, 12.26, and 14.7 μM), and naphthalene acetic acid (NAA) (5.3, 10.60, 15.90, 21.20, and 23.85 μM) were used for the root development. Cultures were incubated under the same conditions as above and rooting percentage, number of roots and root length were recorded after 6 weeks of incubation.

All the experiments were conducted in triplicates and each set of experiment was carried out with 20 explants. Analysis of variance and mean separation was carried out using Duncan’s multiple range tests (DMRT) utilizing the SPS software.

### Hardening and transplantation

Plantlets with properly developed roots were taken out from the culture tubes/flasks after 6-weeks of incubation and washed gently under running tap water to detach the traces of the medium from the roots. Initially to optimize the hardening conditions, the regenerated plantlets were transferred into two set of plastic cups (8×7 cm), one set was filled with a mixture of soil and sand in equal ratio (w/v) while another set was containing only sand. All the plantlets were maintained in the green house at 25±2 °C. Initially to maintain the humidity, plants were covered with transparent polythene sheet and removed after 1 week. After 2 months, plantlets were shifted to nursery black polybags (4.5×8 inch) containing soil and maintained in the poly house. Plantlets were provided half-strength modified Hoagland solution [19] at 3 days interval. In order to acclimatize plants to field conditions, plantlets were transferred to poly bags containing compost enriched soil after 4 weeks and maintained in the open.

### Meiotic studies

For chromosome counts and male meiosis, suitable-sized floral buds were fixed in Carnoy’s fluid (absolute alcohol: chloroform: glacial acetic acid in a ratio of 6:3:1 (v/v)). Samples (BSD 123495) for the study were collected from the plant conserved in the Experimental Botanical Garden. Young and emerging anthers from unopened buds were squashed in 1% acetocarmine and meiotic preparations were made. In each case, 50-100 meiocytes were observed under light microscope at different stages of meiosis for chromosome counts and detailed meiotic course. For microsporogenesis, 100-200 sporads were analyzed in each case. Pollen fertility was assessed through stainability tests by crushing the completely developed anthers in glycerol-acetocarmine mixture (1:1). Well-filled pollen grains with totally stained cytoplasm and nuclei were counted as fertile whereas shriveled and those with stainless/incompletely stained cytoplasm were noted as sterile. Photomicrographs of meiocytes, sporads, and pollen grains were taken from temporary preparations using Nikon microscope fitted with a digital camera.

## Results

### Shoot induction and proliferation

**Fig. 1:**
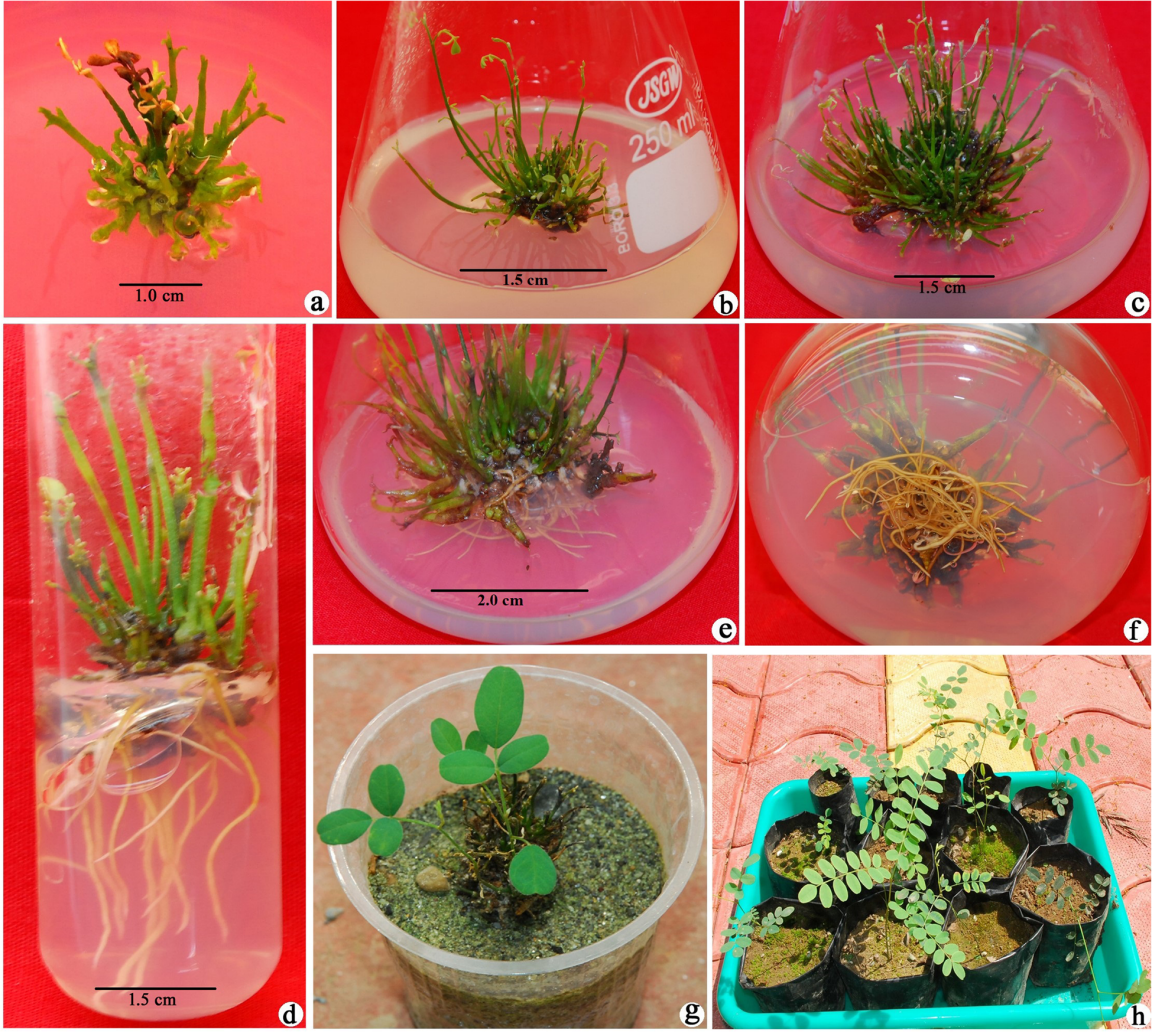
Micropropagation of *Sophora mollis*. **a** Initiation of shoots from shoot tip explants inoculated onto MS medium enriched with BAP (8.9 μM L^−1^) after 14 days of incubation, (**b** and **c**) proliferation of shoots in shoot proliferation medium, (**d**, **e,** and **f**) root induction in half-strength MS medium augmented with NAA (21.20 μM L^−1^) after 6 weeks of incubation, (**g**) properly rooted plantlets transferred to plastic cups containing sand, (**h**) fully acclimatized plants transferred to poly bags containing soil after 4 months of transfer

The excised shoot tip explants inoculated onto the shoot initiation basal MS medium (control) did not show any morphogenic response. When basal media was enriched with BAP (2.2 to 11.1 μM), TDZ (2.27 to 6.8 μM), and kinetin (2.32 to 9.3 μM), a significant increase was observed in shoot formation percentage and maximum 96.27% shoot development was observed in BAP substituted medium followed by TDZ (78.69%) and kinetin (76.78%) (Table 1). Since BAP (8.9 μM), TDZ (4.54 μM), and kinetin (6.9 μM) yielded the maximum shoot proliferation rate in the MS medium, they were further tested in combination with various concentrations of NAA (0.53-2.65 μM). But no significant difference was observed in the shoot proliferation rate, besides shoot formation, callusing was also observed and consequently a reduction in the number of shoots. Based on all the experiments, MS medium supplemented with 8.9 μM BAP was considered optimal for the shoot development and a maximum of 96.27% shoot formation was achieved with 25.32 mean shoot number per culture and 4.5 cm shoot length (Fig. 1a-c).

**Table 1:** Effect of cytokinins and NAA on shoot development from shoot tip explants of *S. mollis* inoculated onto MS medium after 8 weeks of culture

Plant growth regulators (μM)	Explants with shoots (%)	No. of shoots per explant (#)	Shoot length (cm) (#)
MS_0_			
BAP			
2.2	68.24	10.74 ± 0.5^g^	2.5 ± 0.6^d^
4.4	75.81	19.22 ± 0.78^c^	3.5 ± 0.5^bc^
8.9	96.27	25.32 ± 0.83^a^	4.5 ± 0.8^a^
11.1	66.58	20.21 ± 0.8^b^	4.2 ± 0.6^ab^
TDZ			
2.22	54.59	13.22 ± 0.5^f^	1.5 ± 0.69^e^
4.54	78.69	21.54 ± 0.3^b^	3.5 ± 0.4^bc^
6.81	65.38	17.21 ± 0.29^cd^	2.7 ± 0.8^d^
Kinetin			
2.32	53.87	11.15 ± 0.3^g^	2.5 ± 0.78^d^
4.6	68.18	18.69 ± 0.28^cd^	3.5 ± 0.42^bc^
6.9	76.78	19.86 ± 0.36^c^	4.3 ± 0.2^a^
9.3	70.09	17.85 ± 0.3^cd^	4.1 ± 0.39^ab^
BAP + NAA			
8.9 + 0.53	85.98	25.26 ± 0.39^a^	4.1 ± 0.68^ab^
8.9 +1.59	82.21	22.68 ± 1.3^b^	3.9 ± 0.7^b^
8.9 + 2.65	80.23	18.26 ± 0.4^cd^	3.9 ± 0.6^b^
TDZ+NAA			
4.5 + 0.53	78.65	20.21 ± 0.31^c^	2.5 ± 0.46^d^
4.5 +1.59	72.34	18.96 ± 0.42^cd^	2.4 ± 0.4^d^
4.5 + 2.65	69.23	16.17 ± 0.47^de^	2.4 ± 0.41^d^
Kinetin + NAA			
6.9 + 0.53	76.16	19.21 ± 0.71^c^	4.0 ± 0.8^ab^
6.9 +1.59	74.25	17.11 ± 0.29^cd^	3.9 ± 0.7^b^
6.9 + 2.65	68.95	13.24 ± 0.2^f^	3.9 ± 0.9^b^

^#^Data are presented as the mean ±SD (*n*=20)

Means followed by different letter within columns indicate significant differences at *p*≤0.05

### Root induction

Tufts of healthy shoots (4.0 cm height) were shifted for the root induction onto basal MS and modified MS medium (half and quarter strength). Shoots transferred to basal MS medium did not yield any rooting response, while 10.14 and 7.32% rooting was observed in the half and quarter-strength MS medium, respectively. Since half-strength MS medium yielded better rooting response, further experiments were conducted in the half-strength MS medium. By incorporation of IBA (4.9-14.7 μM), NAA (5.3-23.85 μM) and IAA (5.71-22.84 μM) into half-strength MS medium, a significant increase was observed in the rooting percentage. Maximum rooting rates of 86.3, 39.45, and 37.29% were observed in NAA, IBA, and IAA augmented half-strength MS medium (Table 2). The half-strength MS medium, augmented with NAA (21.2 μM) was found to be the optimal for root development in *S. mollis* and 86.3% rooting was achieved with average 21.26 numbers of roots per shoot after 6-weeks of incubation (Fig. 1d-f).

**Table 2:** Effect of auxins on root induction in in vitro regenerated shoots of *S. mollis* in half-strength MS medium after 6 weeks of culture

Auxins (μM)			Rooting (%)	No. of roots per shoot (#)	Root length (cm) (#)
IBA	NAA	IAA			
½ MS	0	0	10.14	1.9± 0.39^j^	0.5± 0.2^g^
4.9	0	0	18.11	4.9± 0.87^i^	1.5± 0.29^f^
7.36	0	0	21.32	7.96± 0.41^fg^	2.7± 0.3^c^
9.8	0	0	27.21	8.85± 0.7^f^	2.8± 0.27^c^
12.26	0		39.45	12.08± 0.71^d^	3.1± 0.4^b^
14.7	0		33.19	10.30± 0.9^e^	3.0± 0.3^b^
0	5.3	0	28.89	12.15± 0.81^d^	1.5± 0.49^f^
0	10.60	0	55.28	15.33± 0.62^c^	2.0± 0.4^e^
0	15.90	0	75.38	18.54± 1.0^b^	4.2± 0.82^a^
0	21.20	0	86.30	21.26± 1.2^a^	4.5± 0.9^a^
0	23.85	0	80.18	19.10± 0.92^b^	4.3± 0.9^a^
0	0	5.71	17.24	5.69± 0.42^h^	1.6± 0.28^f^
0	0	11.42	23.54	8.14± 0.69^f^	2.5± 0.35^cd^
0	0	17.13	37.29	11.23± 0.29^de^	2.9± 0.4^b^
0	0	22.84	31.12	10.21± 0.5^e^	2.7± 0.39^c^

^#^Data are presented as the mean ±SD (*n*=20)

Means followed by different letter within columns indicate significant differences at *p*≤0.05

### Hardening and transplantation

To optimize the hardening conditions, initially twenty plantlets each, with well-developed roots, were shifted to plastic cups containing a mixture of soil and sand in 1:1 ratio (w/v) and sand only (Fig. 1g). All the plantlets were kept inside the greenhouse to adapt to open environment for 1 month. Plantlets shifted to sand responded better and after 2 months plants were shifted to nursery black poly bags containing compost enriched soil in the greenhouse. Fully acclimatized plants were finally transferred to the open environment with 60% success (Fig. 1h), and plants were also transferred to suitable wild habitats under the habitat rehabilitation and species recovery program.

### Meiotic studies

**Fig. 2:**
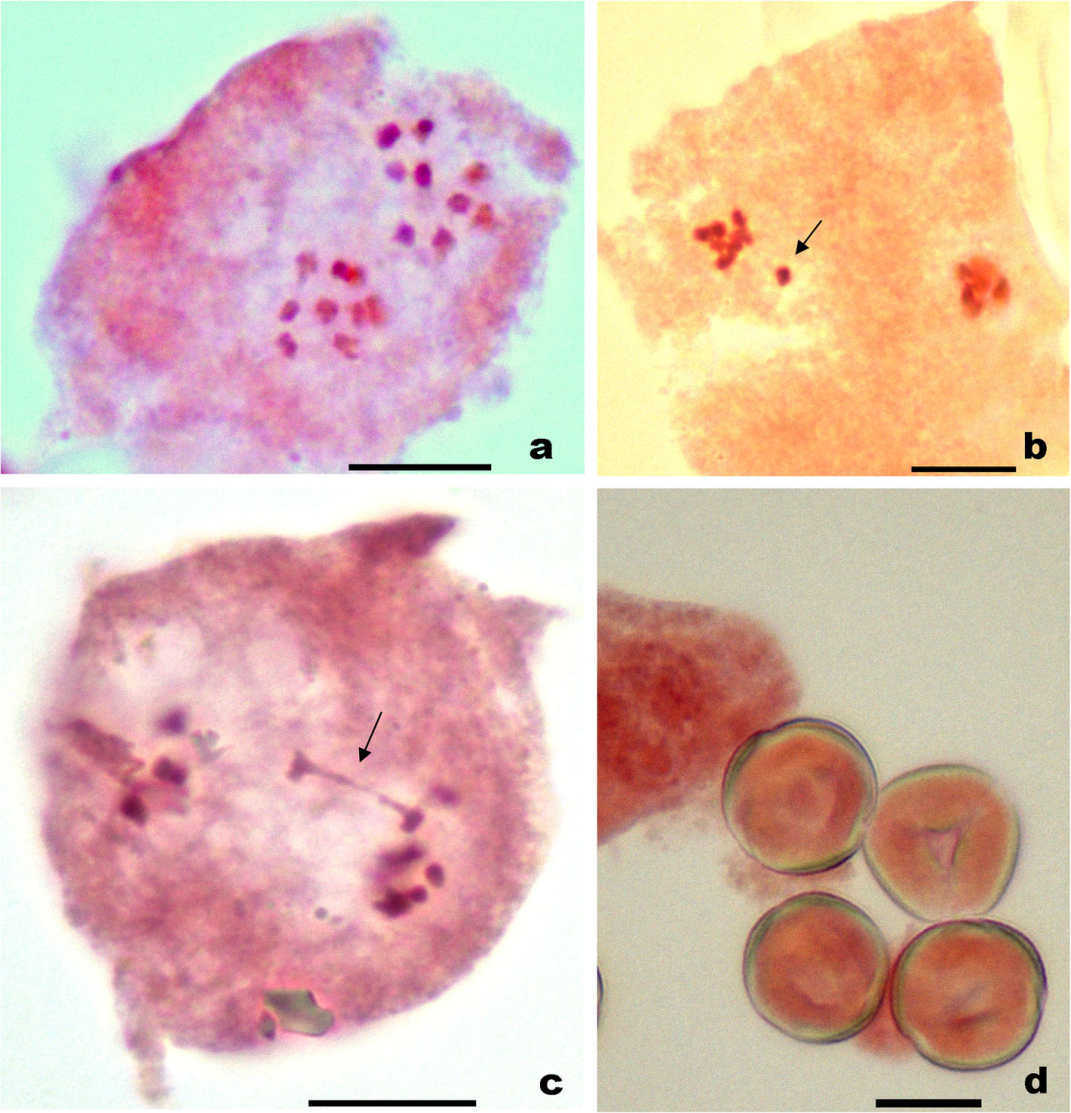
Chromosomal study in *Sophora mollis*. **a** A pollen mother cell showing *n*=9 at metaphase-II, (**b**) lagging of chromosome at anaphase-I (arrowed), (**c**) non-disjunction of chromatin material at anaphase-I (arrowed), (**d**) apparently fertile/stained pollen grains. Scale =10 μm

Meiotic chromosome number of *n* = 9 was determined in *S. mollis* by the presence of 9:9 chromosomes distributions at metaphase-II (Fig. 2a). In very few instances, lagging chromosomes and non-disjunction of chromatin material was observed (Fig. 2b, c). Majority of the pollen mother cells showed normal pairing of chromosomes and equal segregation at anaphase. Consequently, hundred percent pollen fertility was recorded (Fig. 2d).

## Discussion

*S. mollis* is a small deciduous perennial shrub with distinct therapeutic properties and used in traditional Chinese drugs since time immemorial. The unsustainable overexploitation has resulted into the dwindling population size of species in the wild. Thus, plant propagation through tissue culture is recognized as a viable alternative for the multiplication, conservation, and utilization of threatened taxa [20, 21]. Reintroduction of plants into their native environment, under the species recovery and habitat rehabilitation programs, is becoming an increasingly utilized strategy in threatened plant conservation and also proven successful in a variety of species [22, 23].

The in vitro study on *S. mollis* revealed that the MS medium enriched with 8.9 μM BAP was observed to be optimal for the development of shoots and maximum 96.27% shoot formation was achieved with 25.32 ± 0.83 mean shoot number per culture and 4.5 ± 0.8 cm shoot length, respectively. The current finding is in accordance to the previous reports on *Sophora tonkinensis* [24] in which shoot development was observed in MS medium fortified with 2ip (2.0 μM l^−1^) and 5.0 shoots per culture was obtained. While contrary to this, shoot development was also achieved in combination of BAP and auxin (NAA, IBA, and IAA) in *S. tonkinensis* [10], *S. flavescens* [11] and *S. toromiro* [9]*.* Explants inoculated onto MS medium enriched with TDZ and NAA, exhibit hyperhydricity in shoots, thus reducing the total shoot number, and similar phenomenon was also reported in *S. flavescens* [11]*.* Among all the cytokinins used, BAP alone proved to be the most optimal and maximum shoot formation (25.32) was achieved.

Half-strength MS medium augmented with NAA (21.2 μM) was found to be the optimal for root development in *S. mollis.* Maximum 21.26 ± 1.2 roots per shoot were observed with average root length of 4.5 cm after 6-weeks of incubation. NAA was also the most appropriate for root induction in *S. flavescens* [11]. All the in vitro raised plantlets were successfully hardened with 60% of success and were finally transferred to the open environment. Well established plants planted to the wild habitat, under the Habitat Rehabilitation and Species Recovery Program, were also growing successfully and 80% survival rate was reported after 6 months of transfer.

The meiotic chromosome analysis of *S. mollis* exhibit a basic chromosome number of *x* = 9, which is in accordance to previous studies on the species [12–15, 17]. This is the first report of chromosome count in the species from Indian Himalayan region. The species occurred at diploid level (2*x* = 18, with base chromosome number *x* = 9) as per the current study. Further, the meiotic course in the species was found to be normal with hundred percent pollen fertility.

## Conclusion

In conclusion, the current investigation first time describes an efficient and reproducible micropropagation protocol for the *Sophora mollis*, a threatened multipurpose species of the North-West Himalaya*.* This micropropagation system assured effective establishment, mass multiplication, and could offer an in vitro strategy for the ex situ conservation of this threatened shrub. This is the first report of chromosome count in *S. mollis* from Indian Himalayan region and revealed the basic chromosome number *x* = 9.

## Data Availability

Not applicable

## Abbreviations

BAP: 6-Benzylaminopurine
NAA: Naphthalene acetic acid
IBA: Indole butyric acid
IAA: Indole acetic acid
TDZ: N-Phenyl-N0-1,2,3-thiadiazol-5-urea or thidiazuron
